# Processing of Action and Sound Verbs in Context: An FMRI Study

**DOI:** 10.1515/tnsci-2019-0035

**Published:** 2019-10-15

**Authors:** Margot Popp, Natalie M. Trumpp, Markus Kiefer

**Affiliations:** 1 Ulm University, Department of Psychiatry, Ulm, Germany

**Keywords:** embodied cognition, grounded cognition, action-related concepts, sound-related concepts, language, functional magnetic resonance imaging, contextual flexibility

## Abstract

Recent theories propose a flexible recruitment of sensory and motor brain regions during conceptual processing depending on context and task. The present functional magnetic resonance imaging study investigated the influence of context and task on conceptual processing of action and sound verbs. Participants first performed an explicit semantic context decision task, in which action and sound verbs were presented together with a context noun. The same verbs were repeatedly presented in a subsequent implicit lexical decision task together with new action and sound verbs. Thereafter, motor and acoustic localizer tasks were administered to identify brain regions involved in perception and action. During the explicit task, we found differential activations to action and sound verbs near corresponding sensorimotor brain regions. During the implicit lexical decision task, differences between action and sound verbs were absent. However, feature-specific repetition effects were observed near corresponding sensorimotor brain regions. The present results suggest flexible conceptual representations depending on context and task. Feature-specific effects were observed only near, but not within corresponding sensorimotor brain regions, as defined by the localizer tasks. Our results therefore only provide limited evidence in favor of grounded cognition theories assuming a close link between the conceptual and the sensorimotor systems.

## Introduction

Conceptual representations in human longterm memory constitute the meaning of words and are therefore a major prerequisite for language comprehension and production [1, 2]. However, despite the common agreement for conceptual representations being stored in semantic memory [3], it is controversially debated, how concepts are represented within this memory system [2]. According to classical amodal theories, conceptual information is represented in an abstract and symbolic way detached from the sensorimotor brain systems involved in perception and action [4, 5]. These amodal representations are thought to be held in heteromodal brain regions such as the anterior temporal cortex including the temporal pole [6, 7]. Some variants of amodal theories assume that sensorimotor activation occurs as a consequence of semantic elaboration after access to the amodal concept (secondary embodiment) [8]. In contrast, a functional contribution of the sensory and motor brain systems for conceptual representations is postulated by modality-specific accounts (also called: embodied or grounded cognition theories) [9, 10, 11, 12]. Modality-specific theories [13, 14, 15, 16-17] propose that concepts are represented in the respective sensory and motor brain systems depending on the relevance of sensorimotor features for a given concept (concept-inherent feature-relevance). It is thought, that access to conceptual knowledge at least partially reactivates modality-specific representations established during former sensory and motor experiences with the referent [2, 18, 19-20]. This account implicates modality-specific brain activations during conceptual processing at multiple levels involving primary and secondary sensorimotor brain areas [13, 21], similar to brain activity during action and perception [22, 23]. There are several variants of modality-specific theories, which vary depending on the precise relationship between the sensorimotor systems and the conceptual system [for a review, see 24]: While some modality-specific accounts assume an identical neural substrate between conceptual and sensorimotor processing [25, 26], other variants of modality-specific theories do not assume a complete overlap of their neural substrates [22, 27, 28-29]. Instead, depending on task or context, a hierarchy of neural circuits involving modality-specific as well as adjacent multimodal higher-level cortices is assumed to be involved in the processing of conceptual information [28, 30, 31].

Evidence for modality-specific processing of concepts comes from behavioral [32], neuroimaging [13, 17, 33, 34] and electrophysiological studies [35, 36, 37]. For example, a functional magnetic resonance imaging (fMRI) study by Hauk and colleagues showed that action words like *to lick*, *to pick* or *to kick* somatotopically activated motor and premotor brain regions similar to those activated during movements of the tongue, fingers and feet [13]. Moreover, lesion or transcranial magnetic stimulation (TMS) studies underline the functional role of modalityspecific brain areas for conceptual processing [17, 38, 39-40]. Kemmerer et al. [38] reported that brain lesions of the arm- or hand-related part of the left precentral gyrus were specifically associated with impaired knowledge about arm- or hand-related concepts. In a similar way, Trumpp et al. [17] reported specific deficits in the processing of sound concepts after

a focal lesion in auditory association cortex encompassing left posterior superior and middle temporal cortex.

Theories on conceptual representations differ not only with regard to the representation format of concepts, but also with regard to the assumed situational stability vs. flexibility [for a review, see 2]. Originally, conceptual representations were assumed to be stable and thus unaffected by different tasks and contexts [41, 42]. Other, more recent approaches, however, propose flexible conceptual representations [43]: Each word can have different shadings of meaning depending on the respective context. For example, a piano is highly related with sound in the context of a musical event but has a strong weight-related meaning in the context of a move [44]. Some amodal approaches like the distributed semantic network model [5, 6, 7] and the controlled semantic cognition (CSC) framework [45] assume flexible amodal conceptual representations held in anterior temporal cortex, a heteromodal brain region serving as semantic hub. Modality-specific approaches, in contrast, assume a situational recruitment of sensorimotor brain regions, which constitute the concept [23, 33, 46]. A recent neurobiologically inspired model explains conceptual flexibility within modalityspecific brain systems at a mechanistic level [31]: Flexibility based on context-related semantic processing is assumed to depend on pre-activation of specific modality-specific circuits by semantic priming mechanisms [31]. Task-related flexibility, on the other hand, is explained by cortical gain control mechanisms. It is assumed that, depending on task requirements, attention is directed to a certain sensorimotor meaning aspect. This is mechanistically realized by a change of the gain in the corresponding neural circuits, thereby regulating their activity depending on the task at hand [31].

In line with the assumption of flexible modality-specific conceptual representations, behavioral [47], neuroimaging [33, 46] and event-related potential (ERP) studies [46] suggest that conceptual features stored in sensory and motor brain areas are flexibly activated depending on context. For example, the level of activation in corresponding sensory and motor brain areas by visual and action nouns changed depending on which conceptual features were highlighted by a semantic context [46]. Furthermore, several studies show task-related modulations of brain activity during conceptual processing supporting the flexibility assumption. For example, modality-specific effects for verbs were found in semantic decision tasks, where feature-specific information is explicitly retrieved, but not in lexical decision tasks, which probe conceptual retrieval implicitly [48, 49]. Conceptual flexibility may explain why modality-specific effects were inconsistently observed across task and context conditions [20, 46].

Previous studies on feature-specific conceptual representations [50, 51, 52] focused on the comparison between nouns and verbs by assuming that the meaning of nouns is primarily characterized by visual objectrelated conceptual features [53], while verbs are primarily associated with action-related features [54]. However, the meaning of nouns does depend not only on visual, but also on acoustic [22] and action [55] features. Likewise, the meaning of verbs is not only constituted by conceptual action features, but also by visual or acoustic features [56]. For this reason, the processing of conceptual features should not be investigated between word classes, but within the respective word class.

Many studies investigated modalityspecific representations of conceptual information within the word class of nouns. Action nouns (e.g. *hammer*) were found to activate fronto-parietal sensorimotor brain areas that were associated with the use of tools [57]. In a similar way, sound nouns (e.g. *radio*) were found to specifically activate temporal brain regions that are associated with the perception of real sounds [22, 58]. Furthermore, electrophysiological experiments found differential ERPs for action- and soundrelated nouns [36] indicating that different modality-specific neural circuits are involved in conceptual processing of nouns depending on conceptual feature relevance [22].

Several investigations also focused on conceptual processing of concept-inherent feature-specific information within the word class of verbs [13, 59, 60, 61-62]. Popp et al. [56] showed that action- and sound-related verbs (e.g. *to throw* or *to crackle*) elicited differential ERP-effects, which were largely similar to previous ERP findings on action-related and soundrelated nouns with respect to the polarity and time course of ERPs [36, 63]. Differential processing of action- and sound-related verbs in corresponding modal brain regions during a lexical decision task has also been demonstrated in a previous fMRI study (Popp et al., submitted for publication). These findings suggest that different activity in motor and auditory brain regions underlie the differential ERP-effects to action- and sound-related verbs. They indicate that words, whether nouns or verbs, are differentially processed depending on their respective concept-inherent feature relevance as predicted by modality-specific accounts. Popp et al. [56] additionally showed that this feature-specific ERP effects to action- and sound-related verbs depended on the context: The polarity of feature-specific ERP effects for action- and sound-related verbs during a lexical decision task was reversed when these verbs were previously presented together with a context noun during a semantic context decision task.

In several repetition priming studies, deactivation of brain areas, an effect, which has been coined “repetition suppression” [64] has been frequently observed [65, 66, 67]. Repetition was expected to lead to reduced neural activity in the respective modality-specific systems depending on verb category (action vs. sound verbs), but only if the same processes are involved for repeated and unrepeated stimuli. However, if repeated stimuli induce new processes such as additional exemplar recognition or explicit memory retrieval, repetition enhancement occurs in those brain regions that are involved in additional operations [64] including middle frontal, inferior parietal and middle temporal gyrus [68, 69]. However, due to overlap of brain potentials on the scalp surface, the ERP methodology does not allow to determine unequivocally whether these polarity-reversed ERP effects for repeated action- and sound-related verbs were due to repetition suppression or repetition enhancement.

The present fMRI study was conducted to investigate effects of task and context on conceptual processing within the modalityspecific framework and addressed three issues. First, we investigated effects of context via additional contextual cues (context nouns) by identifying brain areas that were activated during the processing of contextually related vs. unrelated noun-verb pairs during an explicit semantic decision task. Second, to investigate task-dependent effects on concept-inherent feature-specific conceptual processing, we compared action- and sound-related verb processing during a semantic context decision task, in which semantic information is explicitly retrieved, with verb processing during a lexical decision task, in which access to conceptual information is implicit. Third, to investigate context via the previous presentation of verbs in an explicit context decision task, we investigated how repetition of action- and sound-related verbs from the previous context decision task modulated conceptual processing of action- and soundrelated verbs in a subsequent lexical decision task. To answer these research questions, we have, to the best of our knowledge, for the first time implemented a study design that allows the investigation of conceptual flexibility depending on context and the task within a modality-specific framework.

The present fMRI study comprised four tasks. In the first task, subjects had to perform a semantic context decision task, in which actionrelated or sound-related verbs were provided either with a semantically related or with a semantically unrelated context noun. The second task was a lexical decision task, in which half of the word set comprised verbs previously presented in the semantic context decision task, while the other half of the stimuli comprised new action- and sound-related verbs. With this experimental paradigm, we were able to identify effects of the task and context on action- and sound-related verb processing to test a flexible activation of conceptual representations. The third and fourth tasks were acoustic and motor localizer tasks to determine auditory and motor regions, respectively. These localizer tasks were administered to test the proposal of modality-specific theories [9, 22, 23, 70] that feature-specific activation occurs in the same or adjacent sensory and motor areas activated during the execution of movements and the perception of sounds, respectively. We expected action verbs to more strongly activate a network of brain areas comprised of frontal and parietal regions (motor cortex, somatosensory cortex, superior parietal cortex) as well as the posterior middle temporal gyrus [Popp et al., submitted for publication; 12, 13, 21, 58, 71] than sound verbs. Sound verbs should activate auditory brain areas within the superior and middle temporal gyrus more strongly than action verbs, as shown in previous investigations on nouns [22, 58]. Furthermore, we predicted feature-specific activation to be stronger during the semantic context decision task than during the lexical decision task, because action and sound features have to be explicitly retrieved during a semantic decision [48, 49, 72].

For the comparison of related versus unrelated noun-verb-pairs during the semantic context decision task, we expected featurespecific activations only for verbs that were preceded by a semantically related context noun, because unrelated noun-verb-pairs activate more diffuse meanings. Furthermore, we expected a general relatedness effect in semantic control regions within inferior frontal, middle temporal, inferior parietal and middle frontal cortex [73, 74], because the retrieval of semantic information is easier with related versus unrelated noun-verb-pairs. Moreover, based on the interpretation of ERP repetition effects in the previous ERP study [56] and in line with earlier research on repetition priming, we expected reduced neural activity for repeated action- and sound-related verbs compared to new verbs in a cortical network supporting word recognition. These areas, assumed to exhibit repetition suppression, are expected to comprise left inferior frontal and inferior temporal language regions [75, 76]. In addition to these general repetition suppression effects, we hypothesized specific repetition suppression effects for action- and sound-related verbs in modality-specific brain systems, which process the task-dependent relevant conceptual feature. Alternatively, not only repetition suppression, but also repetition enhancement could be observed in the present paradigm: It is possible that previous presentation of verbs within the context decision task induces elaborative semantic processing of the words in the subsequent lexical decision task [77]. These additional semantic processes for repeated words could lead to repetition enhancement effects compared to new words, *i.e*. an increase of activity in corresponding brain systems [64]. In addition to general repetition enhancement effects in parts of the language system, elaborative processing of repeated words could also result in an increase of activity within motor and auditory brain areas depending on verb category (*i.e*. action vs. sound verbs).

## Materials and Methods

### Participants

Thirty healthy, right-handed volunteers (mean age: 23.33 years; range: 18 – 35 years; 16 ♀) participated in this study. All participants were native German speakers, had normal or corrected-to-normal vision and no history of neurological or psychiatric disorders. Subjects received expense allowance of 17 Euros. The procedures were approved by the Ethics Committee of Ulm University, and subjects gave written informed consent. Five subjects were excluded from analyses due to reaction times (RTs) deviating two standard deviations (*SD*s) from the sample mean in the lexical decision task or exceeding fMRI motion parameters (translation: +/- 3mm, rotation: +/- 1.5mm). Final analyses included data of 25 participants (mean age: 23.48 years; range: 20 – 35 years; 13 ♀).

### Stimuli

The stimulus set consisted of 40 action verbs and 40 sound verbs that were used in a previous study [56]. Action-related and sound-related verbs were characterized by a high concept-inherent relevance of action and sound features, respectively (conceptual feature relevance). These verbs were selected based on independent conceptual feature ratings, in which verbs were rated according to their conceptual action, acoustic, visual and emotion feature relevance as well as according to familiarity. The selected action- and sound-related verbs had a high relevance of action and sound features, respectively, and a low relevance of most of the other features. Despite careful matching, action-related verbs had significantly higher concept-inherent visual feature relevance compared to sound-related verbs (*p* = .008; *t*(78) = -2.72). Visual features are commonly associated with action execution [56, 78], making the matching of the stimulus material difficult. Stimuli were further matched for familiarity, emotion and for the psycholinguistic parameters word length, word frequency, type frequency, lemma frequency, bigram frequency and trigram frequency (Table 1).

**Table 1:** Matching of conceptual and psycholinguistic stimulus features for action- and sound-related verbs (M (SD)). ** *p* < .001; * *p* < .05 (paired t-test).

	Action verbs	Sound verbs
Action	5.14 (0.42)**	3.03 (0.80)
Sound	1.93 (0.58)	4.97 (0.56)**
Visual	2.74 (0.46)*	2.32 (0.87)
Familiarity	4.00 (0.80)	3.65 (0.81)
Emotion	2.64 (0.79)	2.78 (0.76)
Word length	7.28 (1.67)	7.30 (1.45)
Word frequency	194.98 (405.80)	145.93 (581.85)
Lemma frequency p. Mio.	32.97 (75.06)	23.39 (80.93)
Type frequency p. Mio	7.74 (16.35)	5.19 (20.73)
Character bigram frequency p. Mio.	996151.77 (308584.05)	957488.78 (266070.34)
Character trigram frequency p. Mio.	486060.42 (899993.00)	468888.67 (113753.97)

For the semantic context decision task, each verb was combined with a semantically related context noun (e.g. *ball* – *to throw*/*bell* – *to ring*), which was also used in a previous study [56]. Context nouns, which were combined with action- and sound-related verbs, respectively, were matched for word length (mean (*M*) action verbs = 6.25 (*SD* = 1.95); *M* sound verbs = 6.30 (*SD* = 2.11); *p* = .91) and for their type of relation (action object/object that makes a sound, action instrument/object that is manipulated and thereby elicits sound, action effector/specific organ that elicits sound or situation/situation, where sound occurs) to the critical verb. It was ensured, that reaction times and error rates were comparable across the noun-verb-combinations of the action and sound feature conditions [56]. The stimulus sets for action- and sound-related verbs were split into two matched lists A and B consisting of 20 context-verb pairs each. The verbs of the two lists did not differ regarding their conceptual action or sound feature relevance and psycholinguistic variables (all *p* > .05). Lists A and B were assigned to the same number of participants in a pseudo-randomized fashion and presented in the different repetition conditions of the lexical decision task (for details see below). Additional 20 verbs with high conceptual action feature relevance and 20 verbs with high conceptual sound feature relevance, different from those of the critical word lists, served as distractors and were paired with a semantically unrelated context noun each. Word length of the unrelated context nouns and the respective verbs were matched with those of the critical word sets. Unrelated distractor verbs did also not differ from the critical verbs in most of the psycholinguistic variables (word frequency, type frequency, lemma frequency, character bigram and trigram frequency as well as familiarity) besides a significant higher feature relevance of emotion than action verbs and a significant higher visual feature relevance than sound verbs (both *p* = .03). Thus, the context decision task consisted of 20 action verbs and 20 sound verbs (derived either from list A or from list B) presented with a semantically related context noun as well as 20 action verbs and 20 sound verbs presented with a semantically unrelated context noun (e.g. *cheetah* – *to tidy*/*eggplant* – *to honk*), which served as distractors. Distractor word pairs were the same for all participants.

For the lexical decision task, pseudoverbs were created by substituting one vowel and one consonant of a real verb with another vowel and consonant, respectively, resulting in a pronounceable but meaningless verb. Pseudoverbs, action- and sound-related verbs were matched for word length.

### General Procedures

Participants completed four tasks in the scanner within 50 minutes: Semantic context decision, lexical decision, acoustic localizer and motor localizer. Participants were instructed about each task in detail outside the scanner and additionally briefly before each task in the scanner. Just before starting the examination inside the scanner, participants practiced the semantic context decision task and the lexical decision task on a computer outside the scanner. For practice trials, we used 12 stimuli in each task, which were not used in the main experiment. Given the low difficulty of the tasks, participants only had one practice sequence. Since the practice trials included different stimuli, it is unlikely that repetition effects of the practice trials were transferred to subsequent trials during scanning. Stimuli were delivered and behavioral data were collected using Experimental Runtime System software (BeriSoft, Frankfurt, Germany). Visual stimuli were presented via MR-compatible video glasses (Resonance Technology, Los Angeles, CA, USA) and acoustic stimuli were presented binaurally through MR-compatible headphones (Resonance Technology, Los Angeles, CA, USA). All visual stimuli were presented in white font on a black background with a font size of 16 points. The sound intensity of acoustic stimuli were adjusted individually for each participant, so that they could clearly hear the stimuli (about 70 dB). In both decision tasks participants responded via button press on a response keyboard. All participants performed the tasks in a fixed order: Semantic context decision task, lexical decision task, acoustic localizer and motor localizer. After completing all four tasks, anatomical T1 images were recorded (6 min). Participants were instructed to refrain from moving their head or body during MR scanning.

#### Semantic context decision task (explicit task)

In the semantic context decision task, each trial started with the presentation of a fixation cross for 500 ms followed by the presentation of a context noun for 400 ms. Thereafter, a clear screen was presented for 500 ms, before the verb appeared for 400 ms. Starting with the presentation of the verb, participants had a response window of 1800 ms. Before the next trial started, a jittered intertrial interval (ITI) with a mean duration of 3000 ms (range from 0 – 13200 ms) was inserted. During the ITI, a clear screen was presented. In order to allow for an efficient estimation of the associated hemodynamic responses [79], the sequence of stimulus categories (action, sound or distractor) and the associated ITI were generated in a pseudo-randomized order using OPTSEQ2 (https://surfer.nmr.mgh.harvard.edu/optseq/). The allocation of each word pair to a position within the fixed sequence of experimental conditions was randomized for each participant. Participants responded via button press on a response keyboard and had to decide as quickly and as accurately as possible whether the presented word pair was semantically related or not. They were instructed to press the left button with their right index finger for semantically related word pairs and the right button with their right middle finger for semantically unrelated word pairs. The semantic decision task lasted about 9 minutes and comprised the acquisition of 260 consecutive brain volume images.

#### Lexical decision task (implicit task)

In the lexical decision task (verb/pseudoverb decision), the full set of 40 action verbs and 40 sound verbs was randomly presented along with 80 pseudoverbs. Half of the words had already previously been presented in the context decision task, the other half was new. Counterbalanced across participants, stimulus lists A and B from the semantic context decision task were assigned to the repeated and new conditions, respectively. In each trial, a fixation cross was presented for 500 ms, followed by the presentation of a verb/pseudoverb for 400 ms. Beginning with the presentation of the verb, participants had a response window of 1800 ms. After the verb disappeared, a clear screen was presented. Subsequent to the response window, the screen remained black during the jittered ITI for 3000 ms on average (range from 0 – 11700 ms). ITIs and sequence of the stimulus categories were generated with OPTSEQ2 (https://surfer.nmr.mgh.harvard.edu/optseq/). As in the context decision task, the allocation of each word to a position in the fixed sequence of events was randomized for each participant. Participants had to decide as quickly and accurately as possible, whether the presented word was a real German verb or a meaningless pseudoverb. The duration of the lexical decision task was approximately 14 minutes (435 volumes acquired in total).

#### Acoustic localizer task

The third task was an acoustic localizer task adopted from previous studies [22, 80]. The acoustic localizer task consisted of nine resting and eight sound blocks, which were presented in an alternating order starting and ending with a resting block. Each block lasted 24 s. Sound blocks alternatingly contained 10 natural (e.g. dogbark) or 10 artificial (e.g. carhorn) sounds, respectively. The sounds had a mean duration of 1468 ms (range: 1050 – 1980 ms) and were followed by a jittered pause with a mean duration of 932 ms (range: 383 – 1481 ms). Participants were instructed to close their eyes and attentively listen to the sounds. The duration of the acoustic localizer was approximately 7 minutes (214 brain volume images acquired in total).

#### Motor localizer task

As a fourth task, a motor localizer task was administered. The motor localizer was similarly constructed as the acoustic localizer task: Resting blocks alternated with movement blocks, each block lasting 24 s (214 MR volumes acquired within 7 minutes). The task started with a resting block, which comprised 10 subsequently presented fixation crosses. The duration of each fixation cross was 300 ms, followed by a clear screen with a jittered duration between 1255 ms and 2752 ms (mean jitter time: 2100 ms). During the subsequent movement block, the fixation cross was replaced by two arrows, pointing either to the right or to the left. The arrows represented the respective hand, with which participants had to press an elastic hand training ball. Within one movement block, all arrows were pointing to the same direction. Accordingly, participants had to press the hand-training ball either 10 times with their right hands or 10 times with their left hands. The arrows appeared on the screen for 300 ms. Jitter times were the same as in the resting blocks. Participants were instructed to press the ball during the motor localizer task with a constant strength and to refrain from moving other body parts.

### FMRI data acquisition and analysis

Structural and functional MR images were acquired using a 3-tesla scanning system (Siemens Prisma, Erlangen, Germany) with a 20-channel head coil. Structural high-resolution T1-weighted images were recorded with a MPRAGE sequence (TR = 2300 ms; TE = 2.32 ms; flip angle = 8°; matrix = 256 x 256; field of view (FOV) = 240 mm; voxel size = .9 x .9 x .9 mm). A T2*-weighted single shot gradientecho EPI sequence was used for functional images (TR = 2000 ms; TE = 36 ms; flip angle = 90°; matrix = 76 x 76; FOV = 192 mm, voxel size = 2.5 x 2.5 x 2.5 mm). Thirty-two transversal brain slices were recorded in ascending order. Both the context decision task and the lexical decision task were implemented as eventrelated design, while both localizer tasks were implemented as block design.

FMRI data were preprocessed and statistically analyzed using SPM12 (Wellcome Trust Centre for Neuroimaging, London, United Kingdom) running on MatlabR2013b (The MathWorks, Natick, USA). The participants’ head motion was corrected by rigid-body-transformation during preprocessing. All images of one series were oriented on the first image. Participants’ head motion was corrected for three rotation and three translation parameters. Differences in slice time acquisition were adjusted by slice time correction based on the middle slice. Images were co-registered to structural T1-weighted images for each participant individually. Then, anatomical structures and functional images were normalized to the MNI (Montreal Neurological Institute) template (resampled voxel size: 2 x 2 x 2 mm^3^). Normalized images were smoothed with an isotropic 8 mm FWHM Gaussian kernel. Data were filtered with a high-pass filter (128 Hz), and an autoregressive model was used regarding intrinsic temporal autocorrelation of the image series.

First-level analyses were performed on the subject level with the general linear model. The design matrix of the semantic context decision task in the first-level analysis contained the conditions sound verbs with semantically related context nouns, action verbs with semantically related context noun, distractor sound verbs with semantically unrelated context nouns, distractor action verbs with semantically unrelated context nouns and trials with erroneous responses as regressors. Altogether, the design matrix included four regressors of interest as well as errors and the six motion parameters as nuisance variables.

For the second-level group analysis, a flexible factorial design was specified with subjects as random factor and the four regressors of interest from the first-level analysis. First, for the main effect of semantic relatedness, we calculated the t-contrasts between semantically related verbs versus semantically unrelated verbs (semantic enhancement) and *vice versa* (semantic suppression). To investigate the main effect of feature category, we calculated the t-contrasts between all sound verbs and all action verbs and *vice versa*. Furthermore, we calculated t-contrasts for the interaction between feature category and semantic relatedness (weighting related sound verbs = -1, related action verbs = 1, unrelated sound verbs = 1, unrelated action verbs = -1) and for the reversed interaction semantic relatedness and feature category (weighting: related sound verbs = 1, related action verbs = -1, unrelated sound verbs = -1, unrelated action verbs = 1). In order to breakdown interaction effects, we calculated simple effects of feature category within semantically related and unrelated verbs. We contrasted related action verbs versus related sound verbs, related sound verbs versus related action verbs, unrelated action verbs versus unrelated sound verbs and unrelated sound verbs versus unrelated action verbs. To investigate subtle differential effects, the statistical threshold was set to *p* < .05 uncorrected for multiple comparisons at the cluster level. Cluster-forming voxel threshold was set to *p* < .001 [81]. According to Trumpp et al. [80], a more liberal statistical threshold at the cluster level was applied, because effect sizes in differential contrasts are usually smaller compared with comparisons against baseline, rendering it difficult to obtain effects that survive corrections for multiple comparisons in whole-brain analyses. Clusters surviving corrections for multiple comparisons are marked in the results tables.

The design matrix for the lexical decision task included regressors for repeated sound verbs, new sound verbs, repeated action verbs, new action verbs, pseudoverbs and errors resulting in all together six regressors and six motion parameters. The second-level group analysis of the lexical decision task was specified using a flexible factorial design, containing all 25 subjects as random factor and the four conditions as regressors of interest. In order to investigate the main effect of repetition enhancement and repetition suppression, we calculated the t-contrasts repeated versus new verbs and new versus repeated verbs, respectively. For the main effect feature category, we calculated the t-contrasts all action versus all sound verbs and *vice versa* (all sound vs. all action verbs).

Furthermore, a t-contrast was calculated for the interaction repetition and feature category (weighting: repeated sound verbs = 1, new sound verbs = -1, repeated action verbs = -1, new action verbs = 1) and for the reversed interaction feature category and repetition (weighting: repeated sound verbs = -1, new sound verbs = 1, repeated action verbs = 1, new action verbs = -1).

In order to test for simple repetition effects, the following t-contrasts were calculated for repetition effects within category: repeated action verbs versus new action verbs (repetition enhancement for action verbs), new action verbs versus repeated action verbs (repetition suppression for action verbs), repeated sound verbs versus new sound verbs (repetition enhancement for sound verbs), and new sound verbs versus repeated sound verbs (repetition suppression for sound verbs). To test for simple feature category effects, comparisons within repeated and new verbs contrasted repeated action verbs versus repeated sound verbs, repeated sound verbs versus repeated action verbs, new action verbs versus new sound verbs, and new sound verbs versus new action verbs. At the cluster level, statistical threshold was set to *p* < .05 (uncorrected for multiple comparisons; cluster-forming voxel threshold: *p* < .001).

To investigate the effect of task (explicit or implicit) on feature-specific activations of action- and sound-related verbs using the within-design of the present study, we specified a second-level group comparison with a flexible factorial design containing subjects as random factor and regressors for those action- and sound-related verbs, which occurred in both tasks: We compared activity to semantically related action verbs and semantically related sound verbs from the semantic context decision task (explicit task) with new action- and new sound-related verbs from the lexical decision task (implicit task). We restricted the analyses in the lexical decision task to new action and sound verbs (repeated verbs were excluded), in order to be able to assess task-related effects without the confounding influence of repetition effects. The main effect of type of task was calculated with the t-contrasts explicit versus implicit task and implicit versus explicit task. For the main effect of feature category, we calculated the t-contrasts “action versus sound verbs” and “sound versus action verbs”. Furthermore, t-contrasts for the interaction feature category and task (weighting: sound verbs explicit task: -1; action verbs explicit task: 1; new sound verbs implicit task: 1; new action verbs implicit task: -1) and for the reversed interaction task and feature category (weighting: sound verbs explicit task: 1; action verbs explicit task: -1; new sound verbs implicit task: -1; new action verbs implicit task: 1) were calculated. The statistical threshold was set to *p* < .05 uncorrected for multiple comparisons at the cluster level (cluster-forming threshold at the voxel level: *p* < .001).

For the analyses of the acoustic and motor localizer task, blocks of sound and action events were convolved with the canonical hemodynamic response function together with the six motion parameters (effects of no interest), respectively. Activations of all participants were included in a second-level analyses by one-sample t-test in order to compare event blocks against baseline. Since comparisons against baseline have typically larger effect sizes than more subtle contrasts, a more conservative statistical threshold [81] was applied for the analyses of the localizer tasks. At cluster level, a statistical threshold of *p* < .05, family-wise error (FWE) corrected for multiple comparisons was applied (cluster-forming voxel threshold: *p* < .001). For demonstrating an anatomical overlap of the localizer tasks and the conceptual tasks (semantic decision, lexical decision), activations obtained from the contrast action versus sound verbs and sound versus action verbs from the semantic context decision task and activations of the main effect feature category of the lexical decision task were related to activations of the motor and the acoustic localizer, respectively, similar to earlier work [13, 22, 82]. Anatomical labels for activated brain areas were determined using the SPM Anatomy toolbox [83].

## Results

### Behavioral results

Behavioral data were analyzed by calculating mean RTs of correct responses and mean error rates (ERs). Wrong and omitted responses counted as errors. Outlying reaction times (mean RT at the subject level +/- 2 *SD*) were excluded from analysis (outliers semantic context decision task: 4.54%; outliers lexical decision task: 5.25%). Participants performed the semantic context decision task with a mean ER of 2.85% (*SD* = 2.30%). Repeated measures analysis of variance (ANOVA) with the factors semantic relatedness and feature category on ERs revealed a significant main effect of semantic relatedness (*F*(1,24) = 12.71; *p* = .0016; η^2^*p* = .35). Participants made more errors in the semantically related condition (*M* = 4.40%, *SD* = 5.41%) than in the semantically unrelated condition (*M* = 1.30%, *SD* = 2.43%). There was neither a significant main effect of feature category (*M* related action verbs = 4.60%, *SD* = 5.76%; *M* related sound verbs = 4.20%, *SD* = 5.14%; *M* unrelated action verbs = 1.40%, *SD* = 2.29%; *M* unrelated sound verbs = 1.20%, *SD* = 2.61%; *p* = .71) nor a significant interaction between semantic relatedness and feature category (*p* = .90). An ANOVA on RTs with the factors semantic relatedness and feature category revealed a significant main effect of semantic relatedness (*F*(1,24) = 17.81; *p* = .0003; η^2^*p* = .43). Participants reacted faster to semantically related (*M* = 720 ms; *SD* = 77 ms) than to semantically unrelated word pairs (*M* = 760 ms; *SD* = 70 ms). There was neither a significant main effect of feature category (*M* related action verbs = 724 ms, *SD* = 76 ms; *M* related sound verbs = 716 ms, *SD* = 78 ms; *M* unrelated action verbs = 762 ms, *SD* = 72 ms; *M* unrelated sound verbs = 758 ms, *SD* = 71 ms; *p* = .29) nor a significant interaction between semantic relatedness and feature category (*p* = .69).

In the lexical decision task, participants performed with a mean ER of 3.35% (*SD* = 2.30%). The repeated measures ANOVA on ERs with the factors feature category and repetition showed a significant main effect of feature category (*F*(1,24) = 9.41; *p* = .005; η^2^*p* = .28) and repetition (*F*(1,24) = 6.97; *p* = .014; η^2^*p* = .23). Fewer errors were made for action verbs (*M* = 2.60%; *SD* = 3.27%) than for sound verbs (*M* = 4.40%; *SD* = 3.63%) and for repeated verbs (*M* = 2.40%; *SD* = 4.07%) than for new verbs (*M* = 4.60%; *SD* = 5.13%). The interaction between the factors feature category and repetition was not significant (*p* = .25). The repeated measures ANOVA on RTs with the factors feature category and repetition revealed a main effect of repetition (*F*(1,24) = 26.21; *p* = .00003; η^2^*p* = .52). Participants reacted significantly faster to repeated verbs (*M* = 629 ms; *SD* = 47 ms) than to new verbs (*M* = 652 ms; *SD* = 54 ms). There was neither a significant main effect of feature category (*M* action verbs = 636 ms; *SD* = 52 ms; *M* sound verbs = 644 ms; *SD* = 51 ms) (*p* = .12) nor an interaction between feature category and repetition (*p* = .78).

### Neuroimaging results

#### Semantic context decision task (explicit task)

**Figure 1:**
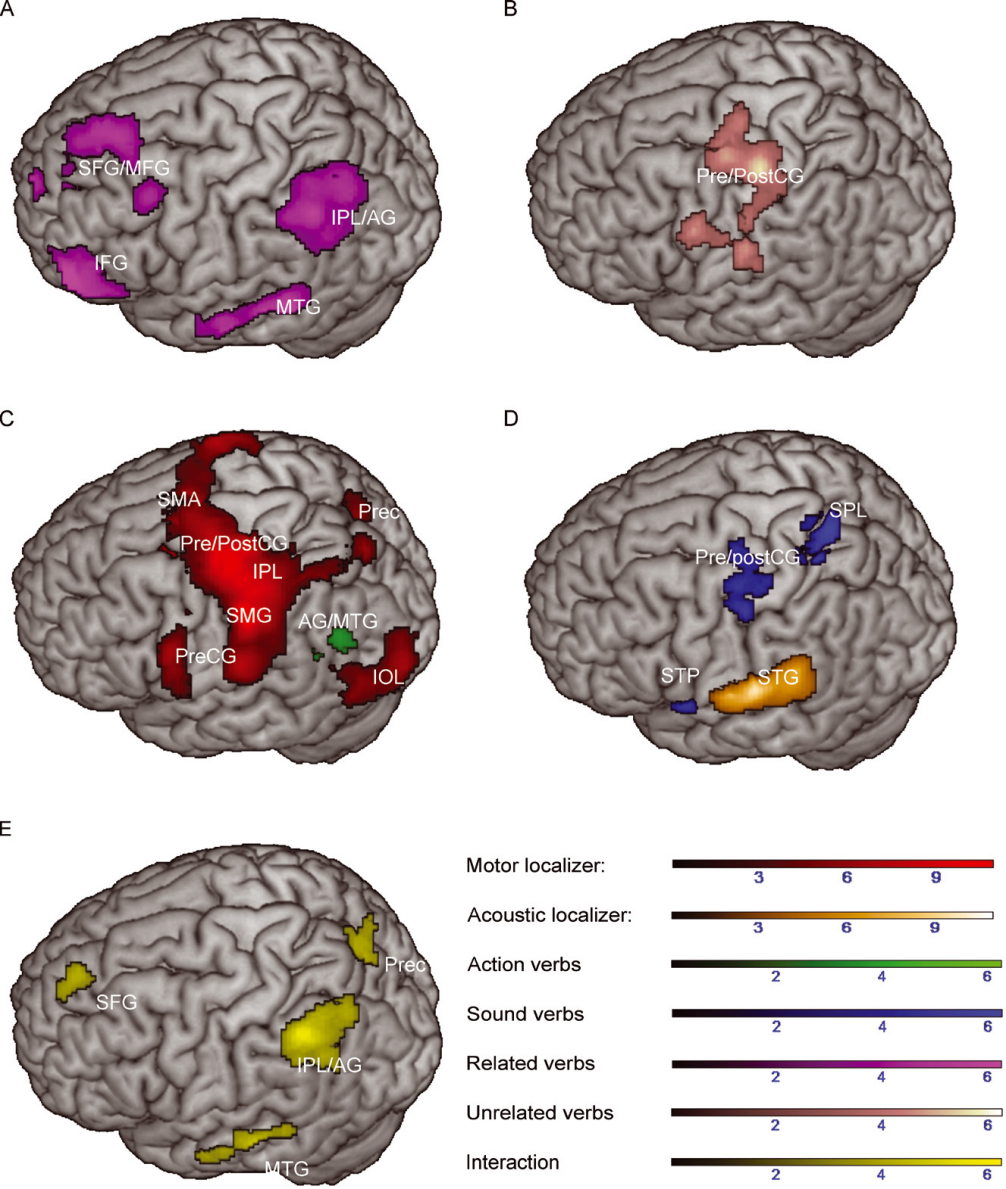
Greater activation to related versus unrelated verbs (A), unrelated versus related verbs (B), action versus sound verbs (C) and sound versus action verbs (D; main contrasts are depicted in magenta, pink, green or blue, respectively) during the context decision task. The main effect of feature category was overlaid with activations during the motor (red) or acoustic localizer (orange), respectively. Color range bars indicate T-scores. Cluster p was set to *p* < .05 (uncorrected) at the cluster level. Results of the motor and acoustic localizer are reported at *p* < .05 FWE-corrected at the cluster-level. AG = angular gyrus; IFG = Inferior frontal gyrus; IOL = Inferior occipital lobe; IPL = Inferior parietal lobe; MFG = Middle frontal gyrus; MTG = Middle temporal gyrus; Prec = Precuneus; Pre-/PostCG = Pre-/Postcentral gyrus; SFG = Superior frontal gyrus; SMA = Supplementary Motor Area; SMG = Supramarginal gyrus; SPL = Superior parietal lobe; STG = Superior temporal gyrus; STP = Superior temporal pole.

In the semantic context decision task, we first calculated the main effect of semantic relatedness. Greater activation to verbs preceded by a semantically related context noun compared with verbs that were preceded by a semantically unrelated context noun was predominantly found in frontal brain regions (middle orbital gyrus, superior medial gyrus and inferior, middle and superior frontal gyri). Greater activation to verbs in the semantically related condition was also observed in middle temporal gyrus, angular gyrus and inferior parietal lobule. Greater activation to verbs in the semantically unrelated condition was found in the supplementary motor area and pre- and post-central gyrus as well as in paracentral lobule. All activations of the main effect of semantic relatedness were located in the left hemisphere (Table 2, Figure 1A, B).

**Table 2:** Peak activations for the main effect semantic relatedness (semantic enhancement and semantic suppression) in the context decision task. Reported are significant results at a statistical threshold *p* < .05 (uncorrected) at the cluster level. Asterisks indicate clusters that are also significant after FWE-correction (*p* < .05). Listed are peak voxels with highest t-values for significant clusters and their local maxima more than 8 mm apart. MNI: Montréal Neurological Institute, FWE: Family-wise error, R: right, L: left.

Brain region	MNI coordinates (mm)	Peak T	Cluster size (voxels)	Cluster p (uncorrected)
Semantic enhancement				
Middle orbital L	-38 48 -10	7.27	585	< .0001*
Inferior frontal pars orbitalis L	-48 42 -6	5.16		
Inferior frontal pars orbitalis L	-52 30 -8	4.29		
Middle temporal L	-64 -42 -6	6.56	509	< .0001*
Middle temporal L	-64 -32 -6	5.65		
Middle temporal L	-64 -14 -18	5.07		
Inferior parietal L	-54 -52 44	5.99	1393	< .0001*
Angular L	-46 -66 48	4.96		
Inferior parietal L	-48 -52 54	4.65		
Superior frontal L	-18 30 56	5.42	1176	< .0001*
Superior medial L	-4 38 34	5.14		
Superior medial L	-6 66 18	4.92		
Middle frontal L	-44 18 44	4.73	167	.003*
Middle frontal L	-34 8 36	3.61		
Semantic suppression				
Supplementary motor area L	-10 -6 52	6.51	206	.001*
Precentral L	-40 -24 58	5.98	965	< .0001*
Precentral L	-38 -10 64	5.08		
Paracentral L	-16 -16 64	4.67		
Postcentral L	-52 -18 22	5.11	180	.002*
Postcentral L	-66 -22 24	3.85		
Precentral L	-58 4 38	4.96	88	.024
Postcentral L	-62 -10 38	3.74		

The analysis of the main effect of feature category revealed greater activation to action verbs in the posterior part of the left middle temporal and angular gyrus compared to sound verbs. Compared to action verbs, sound verbs elicited greater activation in the superior parietal lobule, temporal pole, pre- and postcentral gyrus and in the insula in the left hemisphere (Table 3, Figure 1C, D). The interaction between feature category and semantic relatedness (weighting related sound verbs = -1, related action verbs = 1, unrelated sound verbs = 1, unrelated action verbs = -1) revealed significant activation in left inferior parietal, left middle and superior frontal as well as in left middle temporal brain regions. Further activation was found in the right calcarine sulcus, the right hippocampus, the left precuneus and the thalamus. No significant activations could be observed for the reversed interaction between semantic relatedness and feature category (Table 4, Figure 1E).

**Table 3:** Peak activations for the main effect feature category in the context decision task. Reported are significant results at a statistical threshold *p* < .05 (uncorrected) at the cluster level. Asterisks indicate clusters that are also significant after FWE-correction (*p* < .05). Listed are peak voxels with highest t-values for significant clusters and their local maxima more than 8 mm apart. MNI: Montréal Neurological Institute, FWE: Family-wise error, R: right, L: left.

Brain region	MNI coordinates (mm)	Peak T	Cluster size (voxels)	Cluster p (uncorrected)
Action versus sound verbs				
-	-36 -46 26	5.63	394	< .0001*
Middle temporal L	-46 -52 18	4.44		
Angular L	-42 -64 24	4.11		
-	30 -48 24	4.73	215	.001*
-	18 -36 22	3.98		
-	24 -40 26	3.39		
-	46 -12 -20	4.45	70	.041
Sound versus action verbs				
Superior parietal L	-22 -54 72	4.94	135	.007
Superior parietal L	-32 -48 68	4.45		
Superior parietal L	-18 -44 74	3.42		
Temporal pole superior L	-50 10 -8	4.34	69	.042
Insula L	-40 8 -10	3.65		
Postcentral L	-52 -18 50	4.18	197	.002*
Precentral L	-40 -14 54	3.95		
Precentral L	-32 -14 70	3.83		

**Table 4:** Peak activations for the interaction between feature category and semantic relatedness during the context decision task. Reported are significant results at a statistical threshold *p* < .05 (uncorrected) at the cluster level. Asterisks indicate clusters that are also significant after FWE-correction (*p* < .05). Listed are peak voxels with highest t-values for significant clusters and their local maxima more than 8 mm apart. MNI: Montréal Neurological Institute, FWE: Family-wise error, R: right, L: left.

Brain region	MNI coordinates (mm)	Peak T	Cluster size (voxels)	Cluster p (uncorrected)
Inferior parietal L	-56 -48 48	5.8	999	< .0001*
Inferior parietal L	-50 -54 42	4.77		
-	-34 -48 26	4.74		
-	32 -72 0	4.75	123	.01
Calcarine R	24 -76 6	3.28		
Middle frontal L	-28 12 40	4.7	90	.023
Middle temporal L	-62 -28 -10	4.7	99	.018
Middle temporal L	-62 -40 -4	3.86		
Hippocampus R	26 -20 -14	4.38	69	.042
Precuneus L	-2 -72 44	4.31	309	< .0001*
Precuneus L	-6 -70 54	4.09		
Precuneus L	-8 -64 40	3.81		
Hippocampus R	24 -36 4	4.27	133	.007
Superior frontal L	-14 46 46	4.2	98	.018
Middle temporal L	-54 -6 -26	4.18	79	.031
Middle temporal L	-56 -14 -22	3.8		
Thalamus R	2 -6 4	4.02	142	.006
-	4 0 -8	3.97		
-	8 0 16	3.63		
Thalamus L	-16 -32 12	3.79	72	.039
Thalamus L	-14 -34 4	3.75		

**Figure 2:**
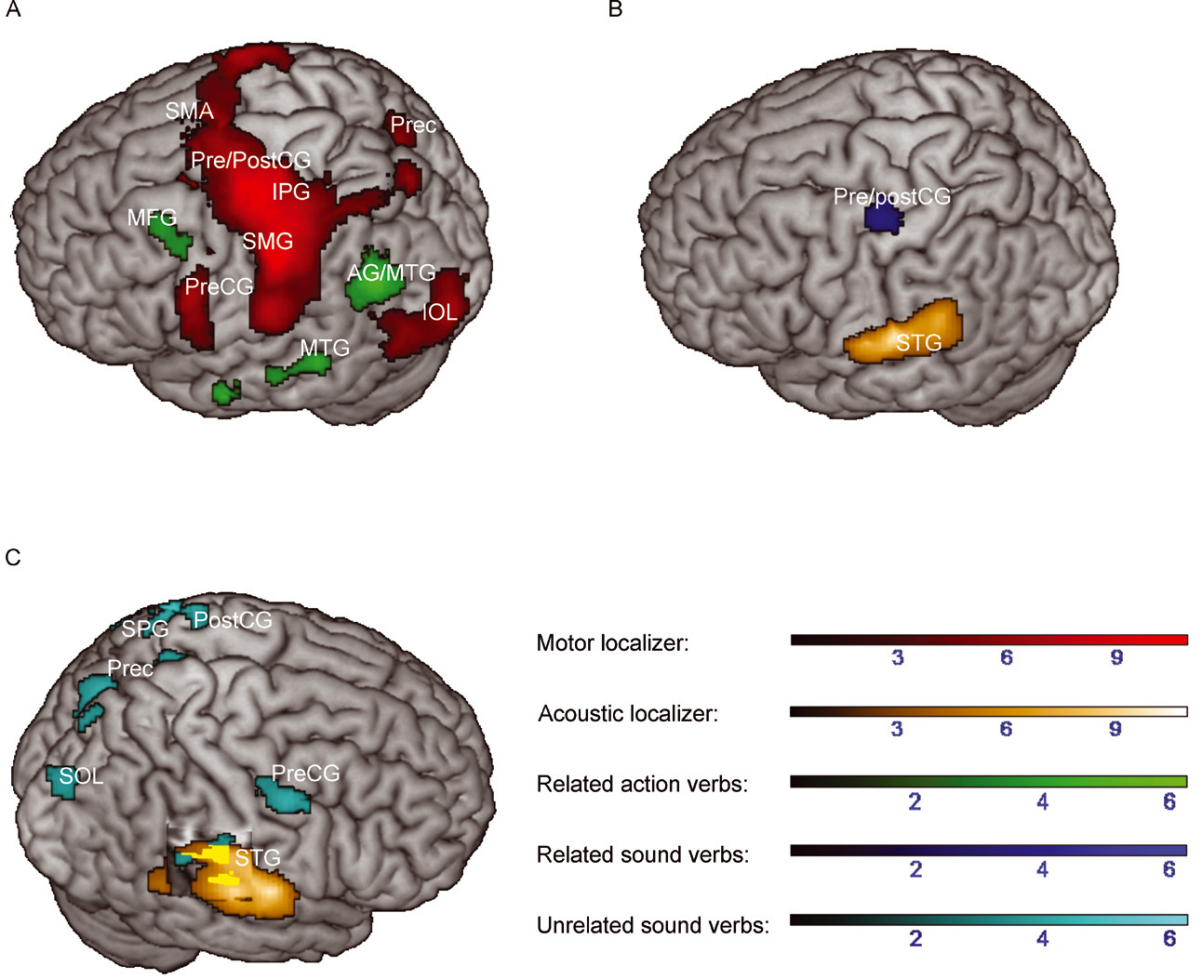
Greater activation to related action versus sound verbs (A), related sound versus action verbs (B) and unrelated sound versus action verbs (C; main contrasts are depicted in green, blue or cyan respectively) during the context decision task overlaid with activations during the motor (red) or acoustic localizer (orange), respectively. Color range bars indicate T-scores. Activations below the surface are visualized by cutout. Cluster p was set to *p* < .05 (uncorrected) at the cluster-level. Results of the motor and acoustic localizer are reported at *p* < .05 FWE-corrected at the cluster-level. AG = Angular gyrus; IOL = Inferior occipital lobe; IPG = Inferior parietal gyrus; MFG = Middle frontal gyrus; MTG = Middle temporal gyrus; Prec = Precuneus; Pre-/PostCG = Pre-/Postcentral gyrus; SMA = Supplementary Motor Area; SMG = Supramarginal gyrus; SOL = Superior occipital lobe; SPG = Superior parietal gyrus; STG = Superior temporal gyrus.

To explore these interactions, we further analyzed simple effects of feature category within semantically related and unrelated verbs. The condition differences reported below were mainly found in the left hemisphere. Greater activation for action verbs compared with sound verbs within related word pairs encompassed the angular gyrus, the anterior part of the inferior temporal gyrus, the posterior part of the middle temporal gyrus, thalamus, hippocampus and the posterior inferior part of the middle frontal gyrus expanding to the precentral gyrus. Greater activation for sound verbs compared with action verbs in the

semantically related condition was observed in the pre- and postcentral gyrus. Within unrelated word pairs, greater feature-specific activation was only found for sound verbs: Unrelated sound verbs activated the left inferior parietal lobule, bilaterally the superior parietal lobule, the left middle cingulate cortex, the right precentral and left postcentral gyrus, the right superior and middle temporal gyrus as well as the right superior occipital gyrus. Furthermore, unrelated sound verbs increased activation in the right hippocampus and putamen and the left amygdala, precuneus, cuneus and lingual gyrus (Table 5, Figure 2).

**Table 5:** Peak activations for the effects of feature category for related and unrelated word pairs during the context decision task. Reported are significant results at a statistical threshold *p* < .05 (uncorrected) at the cluster level. Asterisks indicate clusters that are also significant after FWE-correction (*p* < .05). Listed are peak voxels with highest t-values for significant clusters and their local maxima more than 8 mm apart. MNI: Montréal Neurological Institute, FWE: Family-wise error, R: right, L: left.

Brain region	MNI coordinates (mm)	Peak T	Cluster size (voxels)	Cluster p (uncorrected)
Related action versus related sound verbs				
-	-34 -46 24	6.42	988	< .0001*
Angular L	-40 -52 22	5.57		
Middle temporal L	-42 -62 20	5.29		
Thalamus L	-2 -6 6	5.41	218	.001*
-	2 6 12	4.46		
-	8 -36 8	4.75	147	.005
Inferior temporal L	-54 -8 -28	4.7	92	.022
Middle temporal L	-60 -40 -4	4.36	81	.03
Middle temporal L	-64 -24 -10	3.69		
Hippocampus L	-34 -32 -6	4.36	71	.04
Middle frontal L	-42 18 52	3.9	89	.024
Middle frontal L	-46 12 44	3.54		
Related sound versus related action verbs				
Precentral L	-38 -16 54	4.15	97	.019
Postcentral L	-46 -22 58	3.24		
Unrelated action versus unrelated sound verbs				
-				
Unrelated sound versus unrelated action verbs				
Inferior parietal L	-56 -48 48	5.26	276	< .0001*
Inferior parietal L	-48 -54 54	4.65		
Inferior parietal L	-50 -54 42	4.56		
Hippocampus R	26 -36 0	4.89	98	.018
Amygdala L	-26 -2 -16	4.87	90	.023
-	-20 6 -12	3.55		
Putamen R	26 14 2	4.6	91	.022
Middle cingulate L	-8 -12 42	4.45	204	.001*
Middle cingulate L	-16 -30 44	3.89		
Middle cingulate L	-4 -34 46	3.85		
Precuneus L	-6 -72 52	4.35	279	< .0001*
Cuneus L	-2 -78 38	3.8		
Superior parietal R	10 -78 52	3.72		
Lingual L	-16 -72 -4	4.34	199	.002*
Lingual L	-18 -60 -10	4.34		
Lingual L	-20 -82 -6	3.75		
Precentral R	52 -4 46	4.32	132	.008*
Precentral R	54 4 46	3.8		
Precentral R	52 -14 56	3.78		
Superior temporal R	48 -28 10	4.16	132	.008
Superior temporal R	58 -28 6	3.86		
Middle temporal R	46 -42 10	3.85		
Superior parietal L	-34 -50 66	4.07	103	.016
Postcentral L	-34 -38 70	4.03		
Postcentral L	-26 -36 66	3.98		
Precuneus L	-12 -48 60	4.01	148	.005
Precuneus L	-12 -46 48	3.6		
Superior occipital R	24 -86 30	3.91	66	.046

#### Lexical decision task (implicit task)

**Figure 3:**
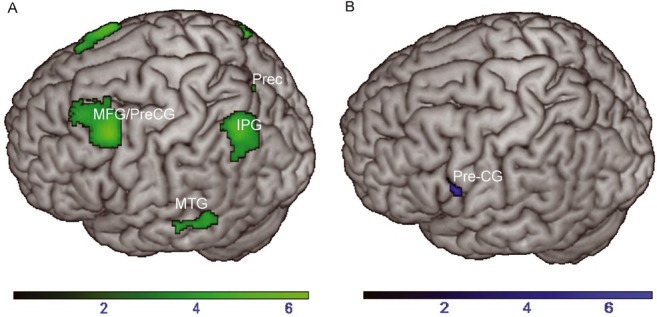
(A) Greater activation to repeated verbs versus new verbs (repetition enhancement) and (B) new verbs versus repeated verbs (repetition suppression) during the lexical decision task. Color range bars indicate T-scores of the respective contrast. Cluster threshold was set to *p* < .05 (uncorrected). IPG = Inferior parietal gyrus; MFG = Middle frontal gyrus; MTG = Middle temporal gyrus; Prec = Precuneus; PreCG = Precentral gyrus.

In a first step, we assessed the main effect of stimulus repetition irrespective of stimulus category: repetition suppression (greater activation for new than for repeated words) and repetition enhancement (greater activation for repeated than for new words). Repetition suppression was found in the right inferior frontal gyrus and in the left precentral gyrus. This cluster expanded from the left precentral gyrus to the left inferior frontal gyrus (Table 6, Figure 3B).

**Table 6:** Activation peaks for the main effect of repetition in the lexical decision task (repetition suppression and repetition enhancement). Reported are significant results at a statistical threshold of *p* < .05 (uncorrected) at the cluster level. Listed are peak voxels with highest t-values for significant clusters and their local maxima more than 8 mm apart. Asterisks indicate clusters that survive cluster FWE-correction (*p* < .05). MNI: Montréal Neurological Institute, FWE: Family-wise error, R: right, L: left.

Brain region	MNI coordinates (mm)	Peak T	Cluster size (voxels)	Cluster p (uncorrected)
Repetition suppression				
Inferior frontal pars triangularis R	46 18 4	5.03	141	.008
Precentral L	-50 6 16	4.42	83	.034
Repetition enhancement				
Middle frontal L	-38 12 46	6.42	575	< .0001*
Middle frontal L	-30 30 52	4.29		
Middle frontal L	-28 16 62	3.49		
Middle frontal R	42 16 54	5.34	313	< .0001*
Middle frontal R	36 12 50	4.86		
Superior frontal R	28 30 54	4.28		
Angular R	44 -62 52	5.33	567	< .0001*
Angular R	40 -60 44	5.2		
Angular R	56 -54 28	3.66		
Inferior parietal L	-34 -60 46	5.13	577	< .0001*
Middle occipital L	-32 -66 36	4.13		
Angular L	-48 -62 42	3.76		
Precuneus L	-6 -68 32	4.93	388	< .0001*
Precuneus L	-6 -68 48	3.86		
Middle cingulate R	6 -40 32	4.08	74	.043
Middle cingulate R	6 -32 34	3.63		
Middle temporal L	-66 -26 -8	4.08	80	.037
Middle temporal L	-66 -36 -4	4.04		
Middle temporal L	-58 -42 -4	4.04		
Middle cingulate L	-12 -42 34	4.01	92	.027

Repetition enhancement was bilaterally observed in the middle frontal gyrus and the angular gyrus. Greater activation to repeated verbs in the left angular gyrus expanded to the middle occipital gyrus and to the inferior parietal gyrus. Repetition enhancement was also obtained in the right superior frontal gyrus, the left middle temporal gyrus, the left precuneus and the mid part of the cingulate gyrus in both hemispheres (Table 6, Figure 3A). Neither the analysis of the main effect of feature category (action vs. sound verbs and *vice versa*) nor the interaction between feature category and repetition yielded suprathreshold voxels. As sound verbs have a considerable conceptual action feature content, sound and action verbs both activate the motor system (see also below). The involvement of the motor system also in sound verbs might decrease the likelihood to find significant interaction effects between repetition and feature category.

**Figure 4:**
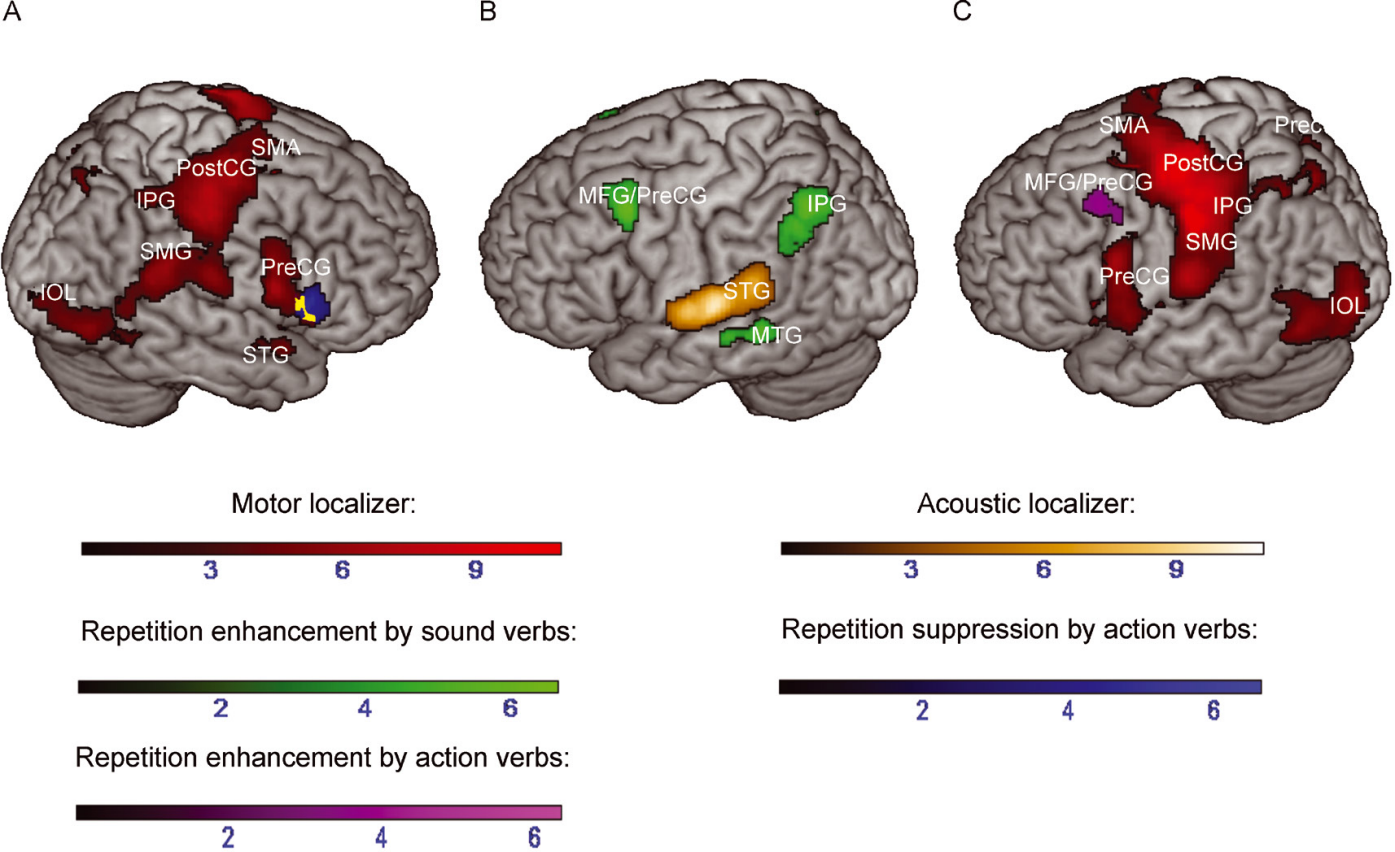
(A) Greater activation to new versus repeated action verbs (blue) (repetition suppression induced by action verbs) overlaid with activations during the motor localizer (red). (B) Greater activations to repeated versus new sound verbs (green) (repetition enhancement induced by sound verbs) overlaid with activations during the acoustic localizer (orange). (C) Greater activations to repeated versus new action verbs (violet) (repetition enhancement induced by action verbs) overlaid with activations during the motor localizer. Color range bars indicate T-scores. Overlapping activations are colored in yellow. Repetition suppression/enhancement effects are reported at *p* < .05 (uncorrected) at the cluster level and results of the motor and acoustic localizer are reported at *p* < .05 (FWE-corrected) at the cluster-level. IOL = Inferior occipital lobe; IPG = Inferior parietal gyrus; MFG = Middle frontal gyrus; MTG = Middle temporal gyrus; Prec = Precuneus; Pre-/PostCG = Pre-/Postcentral gyrus; SMA = Supplementary Motor Area; SMG = Supramarginal gyrus; SPG = Superior parietal gyrus; STG = Superior temporal gyrus.

Furthermore, in order to explore whether action- and sound-related verbs similarly contributed to the main effect of repetition, we analyzed repetition effects separately within each feature category. Repetition suppression for action verbs was found in the right inferior frontal gyrus (Table 7, Figure 4A). For sound verbs, no statistically reliable differences were obtained. Repetition enhancement for action verbs was observed in the left middle frontal and precentral gyrus. Repetition enhancement for sound verbs was bilaterally found in the middle frontal gyrus, the right angular gyrus and in the inferior parietal gyrus in both hemispheres. In addition, repetition enhancement for sound verbs also included an anterior part of the left middle temporal gyrus (Table 7, Figure 4B, C). Comparisons between action- and sound-related verbs calculated separately for repeated and new verbs did not reveal statistically reliable differences.

**Table 7:** Activation peaks for repetition suppression and repetition enhancement by each word category in the lexical decision task. Reported clusters are significant at *p* < .05 (uncorrected) at the cluster level. Listed are peak voxels with highest t-values for significant clusters and their local maxima more than 8 mm apart. Asterisks indicate clusters that survive cluster FWE-correction (*p* < .05). MNI: Montréal Neurological Institute, FWE: Family-wise error, R: right, L: left.

Brain region	MNI coordinates (mm)	Peak T	Cluster size (voxels)	Cluster p (uncorrected)
Repetition suppression by action verbs				
Inferior frontal pars triangularis R	46 20 6	5.74	177	.004*
Repetition suppression by sound verbs -Repetition enhancement by action verbs				
-	-18 44 4	4.33	97	.023
-	-20 46 12	3.62		
-	-16 36 2	3.25		
Middle frontal L	-40 14 46	3.9	85	.032
Precentral L	-50 8 50	3.43		
Repetition enhancement by sound verbs				
Middle frontal R	36 12 50	5.46	294	< .0001*
Middle frontal L	-36 14 46	5.19	255	.001*
Angular R	46 -60 44	4.93	632	< .0001*
Inferior parietal R	54 -54 38	4.19		
Inferior parietal L	-36 -60 46	4.87	389	< .001*
Inferior parietal L	-54 -56 44	4.24		
Inferior parietal L	-44 -52 38	3.9		
Middle temporal L	-60 -42 -4	4.43	101	.021
Middle temporal L	-66 -28 -4	4.1		

### Comparisons between the explicit and the implicit task

The analysis of the main effect of type of task revealed greater activations during the explicit semantic context decision task compared with the implicit lexical decision task in left middle temporal gyrus, the left inferior frontal gyrus, the right superior and left superior medial frontal gyrus, the left supplementary motor area as well as the right pre- and post-central gyrus. Further activations were found in the right middle occipital gyrus, the cerebellum and the right caudate nucleus. Greater activation during the implicit compared with the explicit task was found in the precuneus in both hemispheres, the left middle cingulate gyrus and the left superior temporal gyrus (Table 8).

**Table 8:** Activation peaks for the main effect type of task and main effect feature category across both tasks as well as the interactions between feature type and task. Reported clusters are significant at *p* < .05 (FWE-corrected) at the cluster-level. Listed are peak voxels with highest t-values for significant clusters and their local maxima more than 8 mm apart. Asterisks indicate clusters that survive cluster FWE-correction (*p* < .05). MNI: Montréal Neurological Institute, FWE: Family-wise error, R: right, L: left.

Brain region	MNI coordinates (mm)	Peak T	Cluster size (voxels)	Cluster p (uncorrected)
Explicit versus implicit task				
Middle temporal L	-58 -36 2	11.47	11307	< .0001*
Inferior frontal pars triangularis L	-54 22 24	11.16		
Middle temporal L	-50 -38 0	11.10		
Middle occipital R	28 -98 0	7.00	2411	< .0001*
Cerebellum R	12 -86 -26	6.43		
Cerebellum R	14 -76 -28	5.88		
Posterior-medial frontal L	-4 8 68	6.97	2409	< .0001*
Posterior-medial frontal L	-6 20 60	6.76		
Superior medial L	-4 30 44	6.15		
Precentral R	62 10 26	5.69	86	.028
Superior frontal R	32 -10 68	5.28	213	.001*
-	28 -28 34	5.10	66	.05
Caudate R	18 4 22	4.56	111	.014
-	10 10 18	3.74		
Postcentral R	58 -16 50	4.27	161	.004*
Postcentral R	46 -22 42	3.91		
Postcentral R	54 -16 34	3.47		
Implicit versus explicit task				
Precuneus R	18 -64 30	6.22	2586	< .0001*
Precuneus R	14 -72 44	5.54		
Precuneus L	-8 -78 42	5.40		
Precuneus R	6 -44 50	6.03	865	< .0001*
Mid cingulate L	-12 -38 42	4.03		
Superior temporal L	-48 -14 4	4.23	70	.044
Superior temporal L	-40 -18 -2	3.63		
Action versus sound verbs				
-				
Sound versus action verbs				
-				
Interaction feature category X task				
-	2 4 12	4.89	123	.011
-	0 -4 4	4.04		
-	-34 -44 24	4.81	144	.006
-	-40 -44 14	4.63		
Middle temporal L	-44 -54 18	3.84		
Interaction task X feature category				
-				

Analysis of the main effect of feature category revealed neither significant activations for action versus sound verbs, nor the reversed contrast. An interaction of feature category and task, however, was found in left posterior middle temporal gyrus. This interaction is based on the observation of increased activity to action verbs compared to sound verbs in the explicit task, whereas this feature-specific effect was absent in the implicit task. The reversed interaction did not reach significance (Table 8).

### Localizer tasks

Bilateral hand movements during the motor localizer task bilaterally activated a large fronto-parietal network including pre- and postcentral gyrus, inferior and superior parietal gyrus as well as supplementary motor area with peak activations in the left cingulate cortex, the right supplementary motor area and the left inferior parietal gyrus. Hand movements also activated the left middle and inferior occipital lobe and the right precuneus/cuneus. Binaurally presented sounds during the sound localizer task activated parts of the middle and superior temporal gyrus in both hemispheres with peak activations in the superior temporal gyrus (Table 9).

**Table 9:** Activation peaks for the motor and the acoustic localizer tasks. Reported clusters are significant at *p* < .05 (FWE-corrected) at the cluster-level. Listed are peak voxels with highest t-values for significant clusters and their local maxima more than 8 mm apart. MNI: Montréal Neurological Institute, FWE: Family-wise error, R: right, L: left.

Brain region	MNI coordinates (mm)	Peak T	Cluster size (voxels)	Cluster p (FWE-corrected)
Motor localizer				
Mid Cingulum L	-8 8 38	13.25	24720	< .0001
Supplementary motor area R	6 0 48	12.09		
Inferior parietal L	-54 -24 50	11.83		
Middle occipital L	-36 -90 -2	6.74	1443	< .0001
Inferior occipital L	-44 -68 -10	5.75		
Inferior occipital L	-30 -86 -10	5.19		
Precuneus R	10 -70 46	5.62	183	.04
Cuneus R	14 -66 38	3.85		
Acoustic localizer				
Superior temporal R	54 -10 2	10.95	2221	< .0001
Superior temporal R	64 -24 10	9.50		
Heschls gyrus R	46 -24 10	8.21		
Superior temporal L	-56 -22 6	9.90	1814	< .0001
Superior temporal L	-42 -28 12	9.04		
Superior temporal L	-52 -12 2	8.51		

To test whether activation during the semantic context decision task or during the lexical decision task overlapped with activations during action execution or sound perception, activations in both tasks were related to the activation pattern obtained during the motor and acoustic localizer tasks, respectively. In the semantic context decision task, overlaying significant clusters of the main effect of feature category (action versus sound verbs) with the significant clusters of the motor localizer revealed no overlapping, but adjacent brain activation in the posterior middle temporal gyrus 4 mm apart (smallest distance) (Figure 1C). Overlaying the significant clusters of the contrast sound versus action verbs of the main effect of feature category with the clusters of the acoustic localizer also did not reveal overlapping activation (Figure 1D). Activation to sound verbs was found 12 mm apart from the activations obtained in the acoustic localizer in the anterior portion of the left superior temporal gyrus. Activation by action versus sound verbs was found 4 mm apart from activations obtained during the acoustic localizer in the superior temporal gyrus. Overlaying the significant clusters of the contrast sound versus action verbs with the clusters of the motor localizer revealed overlapping activation in left pre/postcentral gyrus (174 overlapping voxels), superior parietal gyrus (8 overlapping voxels) and superior temporal gyrus (10 overlapping voxels). For simple effects of feature category within semantically related and unrelated verbs, related action versus sound verbs elicited activation in left posterior middle temporal gyrus 4 mm apart from activations of the motor localizer (Figure 2A). Activation to sound verbs within related word pairs did not reveal overlapping activations with the acoustic localizer (Figure 2B). Unrelated sound verbs, however, elicited common activations with the acoustic localizer in the right superior temporal gyrus (93 commonly activated voxels) (Figure 2C).

In the lexical decision task, brain regions showing repetition suppression to action verbs overlapped with activation during the motor localizer in the right inferior frontal gyrus (13 commonly activated voxels) (Figure 4A). Repetition enhancement to sound verbs in the middle temporal gyrus was 16 mm apart from activations obtained during the perception of sounds (Figure 4B). Repetition enhancement induced by action verbs did not overlap with activation during hand movements (Figure 4C). Repetition enhancement induced by action verbs in the left precentral gyrus was 16 mm apart from activations during the motor localizer.

## Discussion

The present study investigated the neural correlates of context effects on action- and sound-related verb processing using fMRI. With our experiments, we wanted to answer three questions: First, are there activation differences to action or sound verbs depending on, whether the verbs are presented together with a semantically related vs. unrelated context noun? Second, can category-specific activation to action- and sound-related verbs similarly be obtained during both explicit (semantic context decision) and implicit tasks (lexical decision)? Third, does the repetition of action- and sound-related verbs, previously presented together with a semantically related context noun in the context decision task, modulate conceptual processing of action- and sound-related verbs in a subsequent lexical decision task?

We observed differential main effects for action- and sound-related verbs only during the explicit semantic context decision task that were only partially in accordance with our hypotheses. These differential category-specific effects for action- and sound-related verbs in the explicit task were more pronounced, when these verbs were presented together with a semantically related context noun. With semantically unrelated context nouns, differences between action- and sound-related verbs were largely absent. During the lexical decision task, differences between action- and sound-related verbs were absent for both new words and words presented repeatedly from the context decision task. However, comparing new with repeated words in the lexical decision task revealed activation differences in a variety of areas: Repetition enhancement (greater activity to repeated vs. new verbs) was obtained in middle frontal, parietal and middle temporal brain regions whereas repetition suppression (greater activity to new vs. repeated verbs) was found in inferior frontal brain regions. Separate analyses of repetition effects in action- and

sound-related verbs indicated category-specific repetition effects.

### Context effects in the semantic context decision task

During the semantic context decision task, we first investigated the neural correlates of processing the verbs in a related versus unrelated context. Stronger activation to semantically related compared to unrelated noun-verb-pairs was found in left inferior parietal, middle temporal, middle frontal and inferior frontal brain areas. Enhanced activation in a fronto-parieto-temporal network subserving semantic retrieval [84] is consistent with findings obtained during semantic priming in previous studies [73, 85, 86]. The same network was also active when the explicit and the implicit tasks were compared in the present study. Hence, as expected, the explicit semantic context decision task more heavily recruited the semantic retrieval network than the lexical decision task. Stronger activation to unrelated compared to related noun-verb-pairs was found in left motor and somatosensory cortex. A comparable activation pattern in primary and premotor areas was found for unrelated compared to related word pairs in a previous study [87]. Greater activation in this brain region to unrelated word pairs could indicate that stimulus inherent action features of the context nouns were more strongly recruited, in order to decide, whether the noun is related to the verb or not. Alternatively, it might be referred to the longer reaction times for unrelated compared with related word pairs. Compared to unrelated word pairs, semantic decisions on related word pairs were faster as it is typically observed in such a task [88]. Although faster reactions were frequently associated with deactivation at the neural level [64], a growing number of studies [73, 85, 86] as well as our results indicate that facilitation at the behavioral level is not necessarily related to decreased neural activity. Instead, behavioral facilitation can also be accompanied by enhanced activation. Most likely, enhanced activation in the semantic retrieval network in the semantically related condition reflect additional semantic processing such as semantic matching of the nouns and the verbs [89].

Independent of semantic relatedness, action verbs elicited greater activation than sound verbs in the posterior part of left middle temporal gyrus, a region that has previously been identified to be involved in the processing of action-related concepts in several neuroimaging studies [12, 58]. These activations in posterior middle temporal gyrus were close to motion-sensitive areas [90, 91] which were also recruited by the performance of movements as shown by the motor localizer (smallest distance: 4 mm) in the present study. Interestingly, recent research indicated that the posterior middle temporal gyrus is functionally connected to the pre- and post-central gyrus [92] underlining its involvement in action processing. Contrary to our expectations, we did not find greater activation to action vs. sound verbs within motor cortex. This is in contrast with previous studies showing activation of primary [e.g. 13, 93] or secondary [e.g. 57] motor areas by action concepts. It should be noted that not all studies on action concepts observed primary or secondary motor cortex activation [94, 95] including a previous study using the same stimulus material (Popp et al., submitted for publication). Possibly, a motor response by pressing a button during the task might have interfered with the processing of conceptual action information. Preparatory processes during active tasks involve neuronal resources of the motor system by eliciting considerable motor activations. This occupancy of the motor system renders it difficult to obtain stronger activation during conceptual processing of dominant action features [96]. This could explain why activations to action words were found in the motor system only during passive tasks [13, 97] which do not require a motor response, but not during active tasks [98]. Therefore, a task that does not require a motor response could possibly yield stronger activations in primary and secondary motor brain areas [96]. Presumably, a comparison with a feature category other than acoustic (e.g. visual) or the comparison with pseudoverbs [84] would also allow to detect stronger activations of the motor system by action verbs: The strong action content of sound-related verbs may have masked the likelihood to find selective motor activations for action-related verbs (see discussion below).

Processing of sound versus action verbs did not yield overlapping, but adjacent (*i.e*. < 15 mm apart) activations with the acoustic localizer. Activity to sound verbs was found outside the auditory cortex and comprised frontal motor areas (primary motor cortex and SMA) as well as somatosensory cortex and parietal visuo-motor areas. These brain regions have been associated with the processing of action-related information [13, 57]. The lack of activation of auditory brain regions on the one hand and the strong motor activations on the other hand might be due to the fact that sound information is less important for the decision, whether the verb and the context noun match compared to action information, even for sound verbs. Consistent with the present results, previous studies on sound-related concepts repeatedly reported the involvement of frontal and parietal brain regions during conceptual processing of sound words [Popp et al., submitted for publication; 22, 36, 63]. We have suggested that sound verbs might have induced activation in motor regions due to their salient action content (Popp et al., submitted for publication). Conceptual feature ratings revealed a relatively high action content in sound verbs, in addition to the high relevance of auditory information. Furthermore, action-sound coupling, *i.e*. sounds are the results of actions, might lead to mutual activation of the auditory and motor systems [99]. We assume that action-sound coupling induces activation within parietal and frontal motor areas through input from the auditory system. This additional input from auditory areas, which was absent in action verbs, may have resulted in stronger activity in various parts of the motor system for sound than for action verbs. It is unlikely that an imbalanced distribution of psycholinguistic parameters across the stimuli was responsible for the observed activation to sound verbs in motor brain regions: The absence of significant differences for action- and sound-related verbs in behavioral performance during the context decision task indicates a successful matching of the critical verbs for psycholinguistic variables and difficulty, although it was not possible to match the visual content of action- and sound-related verbs. Action execution is typically associated with object-related or situational visual features [5], which explains why action concepts not only show a high concept-inherent action feature relevance, but also a considerable high visual feature relevance. For our word material, this imbalance had to be accepted in order to avoid having to resort to untypical word material.

A significant interaction between feature category and semantic relatedness was found in a network of inferior parietal/angular, middle temporal and superior frontal brain regions, which were found to be involved in semantic processing [84]. To explore this interaction, effects of feature category were calculated separately within semantically related and semantically unrelated word pairs. Greater activation to action verbs was found in the middle frontal/precentral gyrus near activations obtained during the motor localizer and located in ventral premotor cortex, a brain region that is associated with the representation of hand actions [100]. Several studies reported activations by action words or sentences involving actions in direct vicinity to those activations obtained in the present study (Arm-related verbs: x: -46, y: 10, z: 40 [101]; Armrelated sentences: x: -54, y: 4, z: 44 [102]; Arm-related sentences: x: -46, y: -2, z: 48 [103]; [for an overview, see 104]). This indicates a recruitment of motor regions by semantically related action verbs. Further activation to related action verbs was found in angular gyrus/posterior middle temporal gyrus, a region that was also activated for action verbs in the comparison between both tasks. As outlined already above, this portion of the middle temporal gyrus is close to activations obtained during the motor localizer, recruited by pictures of tools [105, 106-107] and has been related to action-related motion [90, 91]. Its homologue in the macaque brain involves mirror neurons that respond to action observation and action performance [108]. This suggests that action information is specifically retrieved during the decision, whether a context noun is related to an action verb. The co-activation of more anterior parts of the middle temporal cortex, an area associated with domain-general semantic processing [109], might indicate more detailed semantic retrieval to related action verbs compared with sound verbs.

Greater activation to semantically related sound verbs contrasted with related action verbs was found in motor brain regions within pre-/post-central gyrus in the left hemisphere, which already became visible in the main effect for sound versus action verbs. This indicates that action information is specifically retrieved for semantically related word pairs (see discussion above).

Unrelated sound verbs elicited greater activation than unrelated action verbs in a wide-spread fronto-parieto-occipital visuomotor coordination network including frontal motor cortex, occipital visual areas and parietal motor areas. Further activations to unrelated sound verbs were found in superior and middle temporal brain regions within auditory association cortex in the right hemisphere. These activations considerably overlapped (93 commonly activated voxels) with activations obtained during the perception of sounds during the acoustic localizer task. Activation of auditory association cortex by unrelated sound verbs, but not related sound verbs, suggests that acoustic information is specifically important for the decision that the verb-noun pair is unrelated. Overall, unlike in the related condition, correctly recognizing that the sound-related verb is not related to the context noun seems to depend on retrieval of rich sensorimotor information.

### Repetition effects in the lexical decision task

When comparing repeatedly presented verbs from the context decision task with new verbs during the lexical decision task, we were able to identify general repetition effects on verb processing. Repetition suppression (*i.e*. decreased activation for new versus repeated verbs) was only observed in the inferior frontal gyrus. Repetition suppression in inferior frontal regions has previously been reported in semantic priming [87, 110, 111-112]. It has been assumed that the inferior frontal gyrus mediates semantic analysis of word meaning, which is less effortful for repeated words [110]. Compared to other studies on priming, however, the design of the present study did not comprise an immediate repetition of verbs within a task, but implemented repetition across different tasks. Therefore, the observed suppression effect in the left inferior frontal gyrus can be also related to memory retrieval [73].

Repetition enhancement, *i.e*. larger activity for previously presented verbs compared with new verbs, was observed in a widespread network including middle frontal, middle temporal and parietal cortex. The same enhanced activation pattern of these brain regions was found in several neuroimaging studies on repetition priming of verbal stimuli [68, 73]. Enhanced activation by stimulus repetition in middle frontal, inferior parietal and middle temporal gyrus was associated with the recognition of the earlier presentation of the items [73]. This is in line with activation in posterior temporal, dorsolateral frontal and inferior parietal cortex during the retrieval of explicit memory content [68, 69]. While dorsolateral frontal cortex is associated with memory retrieval [113], posterior temporal and inferior parietal cortex were associated with phonological retrieval processes, which occur automatically during the successful recognition of a verbal item [68]. Thus, enhanced activation in posterior temporal and inferior parietal regions were interpreted as being involved in working memory processes that are associated with explicit judgements about current memory content [68].

In addition to explicit memory retrieval, previously presenting verbs within the context decision task may enhance neural circuits by coding specific aspects of verb meaning or by guiding attention to specific task-relevant conceptual features [69]. As a consequence, sound or action information becomes more strongly salient due to the context noun. This may lead to a semantic enrichment of verb meaning, when the verb is subsequently presented in the lexical decision task. These enriched representations can lead to enhanced neural responses during the repetition of the stimulus in the lexical decision task. These possible processes underlying repetition enhancement effects are discussed in the next paragraph. At the behavioral level, repeated words are typically accompanied by an improvement in reaction times and error rates [69], as seen in the current study.

In order to isolate the specific contribution of action- and sound-related verbs to general repetition effects, repetition effects were analyzed within each feature category. These analyses revealed repetition suppression for action verbs, but not for sound verbs in the right inferior frontal gyrus. Hence, the general repetition suppression effect in this region reported above was mainly driven by action verbs. While the left inferior frontal gyrus is consistently involved in controlled retrieval of semantic knowledge [74], activation of its homologue in the right hemisphere is associated with inhibitory control and the generation of motor responses [114]. Repetition suppression by action verbs in the right inferior frontal gyrus partially overlapped (13 overlapping voxels) with activations obtained during the motor localizer near the precentral gyrus showing that hand movements and conceptual processing of new action verbs partly share their neural substrate. Interestingly, peak activation by new verbs in the right inferior frontal gyrus is close to activations for action verbs found in previous experiments [13]. This also indicates that only a part of the motor system was more active for new compared to repeated action verbs and showed expected deactivation (repetition priming), when meaning aspects of words are repeatedly processed.

However, the majority of areas showed repetition enhancement in both verb categories. Repetition enhancement for action verbs was found in ventral premotor cortex encompassing the middle frontal/precentral gyrus. Repetition enhancement in ventral premotor cortex to action verbs considerably overlapped (31 commonly activated voxels) with the brain region activated by semantically related action verbs in the semantic context decision task. Importantly and in line with our hypotheses, this comparable activation pattern in the context decision task and the lexical decision task suggests that action verbs previously presented together with a semantically related context noun in the context decision task more strongly activated action information in the cortical motor system during the subsequent lexical decision task compared to new action verbs. This is in line with the assumption that the previous presentation of verbs within the context decision task induces elaborative semantic processing of their meaning in the subsequent lexical decision task [64], resulting in enhanced activations for repeated verbs compared to new verbs.

Repetition enhancement for sound verbs was found in middle frontal (this cluster is located more medially than the one for action verbs), inferior parietal and middle temporal brain regions, areas involved in explicit memory retrieval as discussed above. Importantly, repetition enhancement for sound verbs was also found in the left middle temporal gyrus in a part somewhat more anteriorly located than the region recruited by action verbs in the present study. This region corresponds to activations previously found for sound nouns [22, 58]. Repetition enhancement for sound verbs in left middle temporal gyrus indicates activation of conceptual sound information during the lexical decision task. As mentioned before, middle frontal gyrus and inferior parietal areas are associated with memory retrieval processes.

Taken repetition enhancement effects by action- and sound-related verbs together, these findings indicate a recruitment of motor and auditory areas during the lexical decision task, but only when verbs were previously presented in the semantic context decision task. Presumably, presenting the verbs within a fitting semantic context enriches their meaning by triggering elaborative semantic processing. This, in turn, boosts activation of relevant sensory and motor features, when the verb is subsequently presented in the lexical decision task. These putative processes underlying the present repetition enhancement effects are consistent with the assumption of Segaert et al. [69] that multiple processes contribute to repetition enhancement effects: In addition to explicit episodic memory retrieval, attention to specific stimulus features (here: conceptual action or sound information) or the enhancement of functional neural circuits (here: linking action or sound information more strongly to a given concept) might give rise to the observed repetition enhancement effects within auditory and motor areas.

### Comparisons between the explicit and the implicit task

The comparison of activations obtained during the implicit lexical decision task with activations during the explicit semantic context decision task revealed greater activations during the explicit task in a large fronto-parieto-temporal semantic retrieval network [84], which is largely comparable to those activations obtained in the main effect semantic relatedness during the semantic context decision task. Greater activations to the implicit compared with the explicit task were found in a parietal brain region comprising the precuneus, the cingulate cortex and the superior temporal cortex. These brain regions were associated with implicit word processing in previous studies [115]. These results indicate that the explicit task recruited a semantic retrieval network more strongly than the implicit task. Significant interaction of feature category and task were found in left posterior middle temporal cortex. This indicates that action features of verbs were more strongly recruited in the explicit than in the implicit task (see also discussion above).

### Implications for theories of conceptual representations

During the explicit semantic context decision task, we found differential main effects of feature category for action- and sound-related verbs. However, anatomical localization of these activations were only partially compatible with our expectations given the stronger activation of motor brain regions to sound- compared with action-related verbs. During the lexical decision task, category-specific effects for action- and sound-related verbs were observed only in terms of repetition effects. Contrary to our expectations and incompatible with the basic assumptions of any variant of modality-specific theories [13, 14, 15, 16], newly presented action- and sound-related verbs in the lexical decision task did not elicit differential brain activations. Similar to the present results, sensorimotor activation during conceptual processing has previously been mainly observed during explicit semantic tasks [48, 49]. Generally, such task-specific sensorimotor activation has been typically interpreted within amodal theories, which assume that sensorimotor activation occurs only as a consequence of semantic elaboration after access to the amodal concept (secondary embodiment) [8]. A limitation of the present work are the lenient statistical thresholds applied to assess task and feature-specific effects. This indicates that effect sizes were small, and results were statistically not very robust. It should be noted that more lenient statistical thresholds were also applied in earlier studies [80] to capture subtle differential contrasts with small effect sizes. The missing robustness of the effects and the observation of verb category effects on sensorimotor activity in the explicit semantic context task and of different repetition effects as a function of category in the lexical decision task thus seem to favor amodal theories.

However, we think that it is premature to take the present results as well as comparable earlier findings as falsification of modality-specific theories. Firstly, it should be noted that earlier studies observed sensorimotor activity during a lexical decision task for both nouns [22, 58] and verbs (Popp et al., submitted for publication). Secondly, ERP recordings revealed an early onset of sensorimotor activity [14, 36, 56, 116] indicating that it reflects rapid access to modality-specific concept-inherent features and not post-conceptual elaboration processes [77]. Thirdly, it should be noted that the number of stimuli per condition (20 verbs) was low in the present experiments (*i.e*. half of the stimuli used in the previous related work (Popp et al., submitted for publication), in order to be able to investigate repetition effects in a within-subject design. The relatively low signal-to-noise ratio in the present study could have prevented to detect weak sensorimotor activation in the lexical decision task. The number of used stimuli for each category in the present study was the same as in a previous study, which also found no differential feature-specific effects between hand- and foot-related action verbs in a lexical decision task [48]. Fourthly, task and context effects on sensorimotor activation observed in the present study are compatible with the notion of conceptual flexibility within a modality-specific framework [20, 31, 33, 43, 46]. According to this view, the featural composition of a concept strongly depends on the context. Therefore, contribution of specific sensory and motor modalities to a concept is not situationally invariant, but is modulated by task demands or context. This explanation is also compatible with the fact that some studies reported differential effects for verbs with a concept-inherent focus on specific features such as action during implicit tasks [62, 117]: Interestingly, these previous studies included more difficult implicit tasks (e.g. speeded lexical decision or additional hand-movements during the lexical decision). We assume that task orientation (explicit context decision vs. implicit lexical decision task) and the previous presentation of verbs together with a context noun during a semantic context decision task enhances conceptual processing within sensorimotor areas during a subsequent lexical decision task. Future studies with a higher number of stimuli and thus a better signal-to-noise ratio could further test the notion of modality-specific flexible conceptual processing. Finally, the lack of a substantial functional anatomical overlap between conceptual feature activation during the semantic tasks and activation during the acoustic or motor localizer seem to contradict modality-specific theories. Please note that some variants of modality-specific theories [20, 22, 23, 118] do not assume a complete neuroanatomical overlap of activation during the processing of concept-inherent features and neural circuits associated with real action or perception. Re-activation of sensorimotor experiences during conceptual processing may also involve higher-level modality-specific or multimodal association cortex within a hierarchy of functional brain networks at various levels of abstractions, which were not activated by the localizer tasks [28, 30, 31]. It is therefore possible that the functional-anatomical overlap between conceptual and sensorimotor processing depends on the stimuli and actions used in the localizer tasks.

To conclude, in the present study we investigated underlying neural circuits during the processing of action- and sound-related verbs in different tasks and contexts. We found different activation patterns for action- and sound-related verbs depending on task and context near those brain areas that are activated during the execution of movements or the perception of sounds, respectively. These effects only occurred during the explicit retrieval of conceptual information in a semantic context decision task. In the lexical decision task, repetition effects involved specific sensorimotor systems depending on verb category. The present results are in line with variants of amodal theories of conceptual cognition, which propose secondary sensorimotor processing during a semantic elaboration stage after the amodal concept had been accessed [8]. Alternatively, our findings are also compatible with the notion of flexible modality-specific conceptual representations. According to the latter view, sensory and motor features constituting the concept are partially activated depending on context and task demands. Further studies are needed to explicitly examine the factors underlying the strong activation of sound verbs within the motor system and the mechanisms leading to repetition enhancement effects. The present investigation on the processing of action- and sound-related verbs within different tasks and contexts only provides correlative information about the functional neuroanatomy. The functional relevance of the sensorimotor systems for flexible feature-specific conceptual processing could be investigated in future behavioral interference or TMS studies [40, 119].
